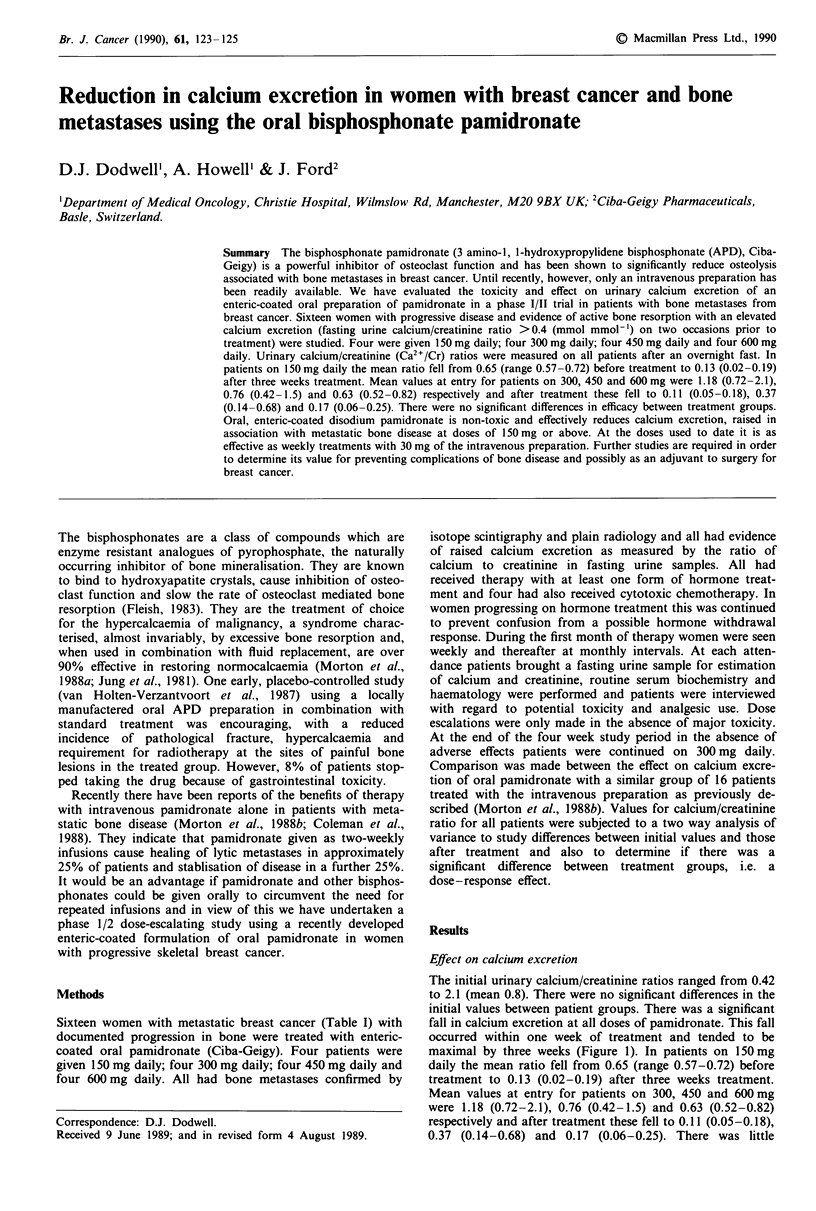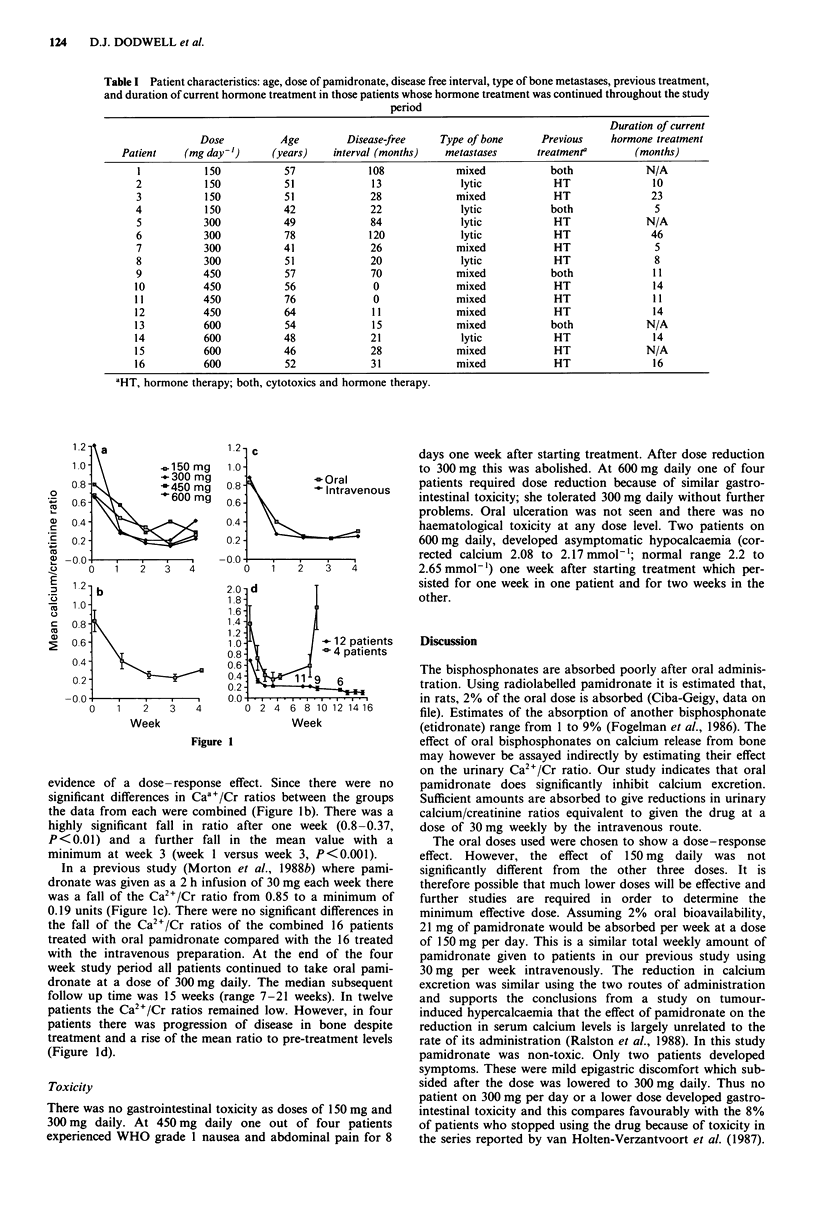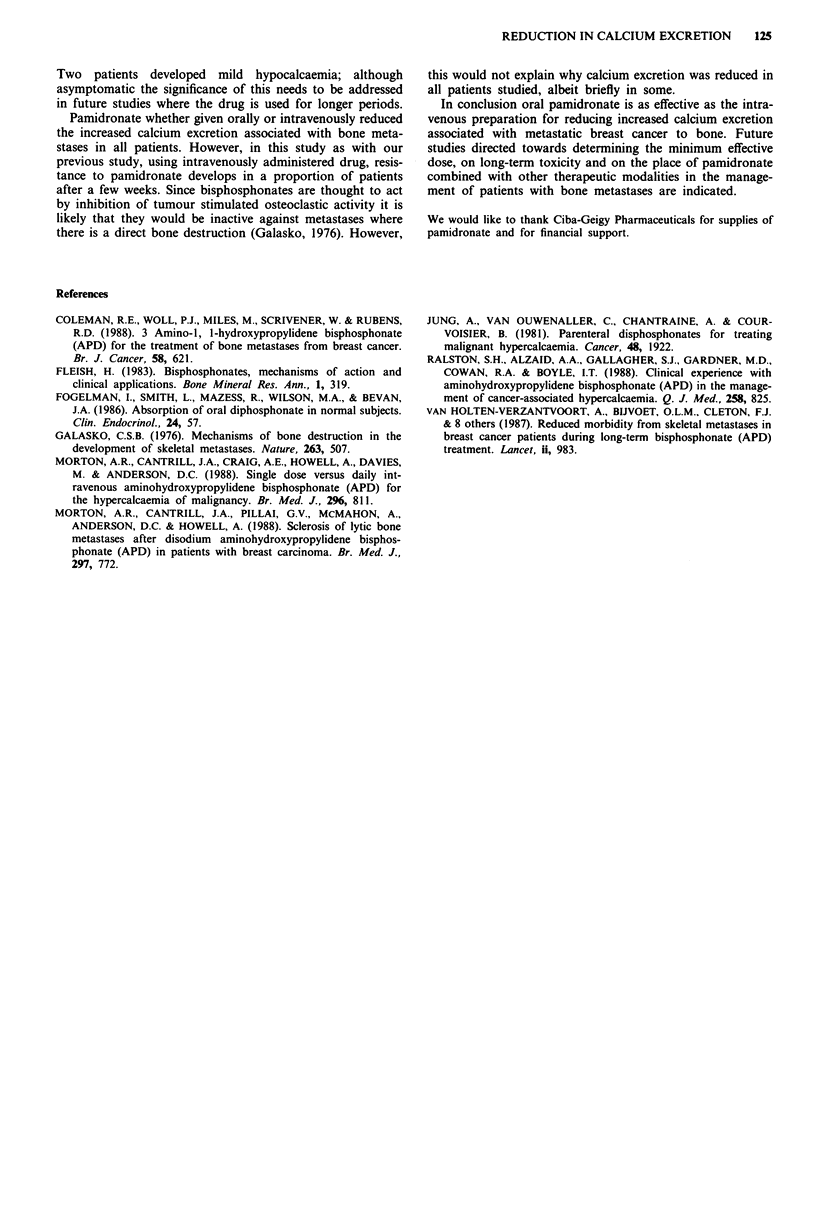# Reduction in calcium excretion in women with breast cancer and bone metastases using the oral bisphosphonate pamidronate.

**DOI:** 10.1038/bjc.1990.25

**Published:** 1990-01

**Authors:** D. J. Dodwell, A. Howell, J. Ford

**Affiliations:** Department of Medical Oncology, Christie Hospital, Manchester, UK.

## Abstract

The bisphosphonate pamidronate (3 amino-1, 1-hydroxypropylidene bisphosphonate (APD), Ciba-Geigy) is a powerful inhibitor of osteoclast function and has been shown to significantly reduce osteolysis associated with bone metastases in breast cancer. Until recently, however, only an intravenous preparation has been readily available. We have evaluated the toxicity and effect on urinary calcium excretion of an enteric-coated oral preparation of pamidronate in a phase I/II trial in patients with bone metastases from breast cancer. Sixteen women with progressive disease and evidence of active bone resorption with an elevated calcium excretion (fasting urine calcium/creatinine ratio greater than 0.4 (mmol mmol-1) on two occasions prior to treatment) were studied. Four were given 150 mg daily; four 300 mg daily; four 450 mg daily and four 600 mg daily. Urinary calcium/creatinine (Ca2+/Cr) ratios were measured on all patients after an overnight fast. In patients on 150 mg daily the mean ratio fell from 0.65 (range 0.57-0.72) before treatment to 0.13 (0.02-0.19) after three weeks treatment. Mean values at entry for patients on 300, 450 and 600 mg were 1.18 (0.72-2.1), 0.76 (0.42-1.5) and 0.63 (0.52-0.82) respectively and after treatment these fell to 0.11 (0.05-0.18), 0.37 (0.14-0.68) and 0.17 (0.06-0.25). There were no significant differences in efficacy between treatment groups. Oral, enteric-coated disodium pamidronate is non-toxic and effectively reduces calcium excretion, raised in association with metastatic bone disease at doses of 150 mg or above. At the doses used to date it is as effective as weekly treatments with 30 mg of the intravenous preparation. Further studies are required in order to determine its value for preventing complications of bone disease and possibly as an adjuvant to surgery for breast cancer.


Br. J. Cancer (1990), 61, 123–125 © Macmillan Press Ltd., 1990

### Reduction in calcium excretion in women with breast cancer and bone metastases using the oral bisphosphonate pamidronate

D.J. Dodwell[1], A. Howell[1] & J. Ford[2]

[1]*Department of Medical Oncology, Christie Hospital, Wilmslow Rd, Manchester, M20 9BX UK;* [2]*Ciba-Geigy Pharmaceuticals, Basle, Switzerland.*

**Summary** The bisphosphonate pamidronate (3 amino-1, 1-hydroxypropylidene bisphosphonate (APD), Ciba-Geigy) is a powerful inhibitor of osteoclast function and has been shown to significantly reduce osteolysis associated with bone metastases in breast cancer. Until recently, however, only an intravenous preparation has been readily available. We have evaluated the toxicity and effect on urinary calcium excretion of an enteric-coated oral preparation of pamidronate in a phase I/II trial in patients with bone metastases from breast cancer. Sixteen women with progressive disease and evidence of active bone resorption with an elevated calcium excretion (fasting urine calcium/creatinine ratio >0.4 (mmol $mmol^{-1}$) on two occasions prior to treatment) were studied. Four were given 150 mg daily; four 300 mg daily; four 450 mg daily and four 600 mg daily. Urinary calcium/creatinine ($Ca^{2+}/Cr$) ratios were measured on all patients after an overnight fast. In patients on 150 mg daily the mean ratio fell from 0.65 (range 0.57–0.72) before treatment to 0.13 (0.02–0.19) after three weeks treatment. Mean values at entry for patients on 300, 450 and 600 mg were 1.18 (0.72–2.1), 0.76 (0.42–1.5) and 0.63 (0.52–0.82) respectively and after treatment these fell to 0.11 (0.05–0.18), 0.37 (0.14–0.68) and 0.17 (0.06–0.25). There were no significant differences in efficacy between treatment groups. Oral, enteric-coated disodium pamidronate is non-toxic and effectively reduces calcium excretion, raised in association with metastatic bone disease at doses of 150 mg or above. At the doses used to date it is as effective as weekly treatments with 30 mg of the intravenous preparation. Further studies are required in order to determine its value for preventing complications of bone disease and possibly as an adjuvant to surgery for breast cancer.

The bisphosphonates are a class of compounds which are enzyme resistant analogues of pyrophosphate, the naturally occurring inhibitor of bone mineralisation. They are known to bind to hydroxyapatite crystals, cause inhibition of osteoclast function and slow the rate of osteoclast mediated bone resorption (Fleish, 1983). They are the treatment of choice for the hypercalcaemia of malignancy, a syndrome characterised, almost invariably, by excessive bone resorption and, when used in combination with fluid replacement, are over 90% effective in restoring normocalcaemia (Morton *et al.*, 1988*a*; Jung *et al.*, 1981). One early, placebo-controlled study (van Holten-Verzantvoort *et al.*, 1987) using a locally manufactered oral APD preparation in combination with standard treatment was encouraging, with a reduced incidence of pathological fracture, hypercalcaemia and requirement for radiotherapy at the sites of painful bone lesions in the treated group. However, 8% of patients stopped taking the drug because of gastrointestinal toxicity.

Recently there have been reports of the benefits of therapy with intravenous pamidronate alone in patients with metastatic bone disease (Morton *et al.*, 1988*b*; Coleman *et al.*, 1988). They indicate that pamidronate given as two-weekly infusions cause healing of lytic metastases in approximately 25% of patients and stablisation of disease in a further 25%. It would be an advantage if pamidronate and other bisphosphonates could be given orally to circumvent the need for repeated infusions and in view of this we have undertaken a phase 1/2 dose-escalating study using a recently developed enteric-coated formulation of oral pamidronate in women with progressive skeletal breast cancer.

### Methods

Sixteen women with metastatic breast cancer (Table I) with documented progression in bone were treated with enteric-coated oral pamidronate (Ciba-Geigy). Four patients were given 150 mg daily; four 300 mg daily; four 450 mg daily and four 600 mg daily. All had bone metastases confirmed by isotope scintigraphy and plain radiology and all had evidence of raised calcium excretion as measured by the ratio of calcium to creatinine in fasting urine samples. All had received therapy with at least one form of hormone treatment and four had also received cytotoxic chemotherapy. In women progressing on hormone treatment this was continued to prevent confusion from a possible hormone withdrawal response. During the first month of therapy women were seen weekly and thereafter at monthly intervals. At each attendance patients brought a fasting urine sample for estimation of calcium and creatinine, routine serum biochemistry and haematology were performed and patients were interviewed with regard to potential toxicity and analgesic use. Dose escalations were only made in the absence of major toxicity. At the end of the four week study period in the absence of adverse effects patients were continued on 300 mg daily. Comparison was made between the effect on calcium excretion of oral pamidronate with a similar group of 16 patients treated with the intravenous preparation as previously described (Morton *et al.*, 1988*b*). Values for calcium/creatinine ratio for all patients were subjected to a two way analysis of variance to study differences between initial values and those after treatment and also to determine if there was a significant difference between treatment groups, i.e. a dose–response effect.

Correspondence: D.J. Dodwell.
Received 9 June 1989; and in revised form 4 August 1989.

### Results

### *Effect on calcium excretion*

The initial urinary calcium/creatinine ratios ranged from 0.42 to 2.1 (mean 0.8). There were no significant differences in the initial values between patient groups. There was a significant fall in calcium excretion at all doses of pamidronate. This fall occurred within one week of treatment and tended to be maximal by three weeks (Figure 1). In patients on 150 mg daily the mean ratio fell from 0.65 (range 0.57–0.72) before treatment to 0.13 (0.02–0.19) after three weeks treatment. Mean values at entry for patients on 300, 450 and 600 mg were 1.18 (0.72–2.1), 0.76 (0.42–1.5) and 0.63 (0.52–0.82) respectively and after treatment these fell to 0.11 (0.05–0.18), 0.37 (0.14–0.68) and 0.17 (0.06–0.25). There was little

**Table I** Patient characteristics: age, dose of pamidronate, disease free interval, type of bone metastases, previous treatment, and duration of current hormone treatment in those patients whose hormone treatment was continued throughout the study period

| Patient | Dose (mg day$^{-1}$) | Age (years) | Disease-free interval (months) | Type of bone metastases | Previous treatment[a] | Duration of current hormone treatment (months) |
|---|---|---|---|---|---|---|
| 1 | 150 | 57 | 108 | mixed | both | N/A |
| 2 | 150 | 51 | 13 | lytic | HT | 10 |
| 3 | 150 | 51 | 28 | mixed | HT | 23 |
| 4 | 150 | 42 | 22 | lytic | both | 5 |
| 5 | 300 | 49 | 84 | lytic | HT | N/A |
| 6 | 300 | 78 | 120 | lytic | HT | 46 |
| 7 | 300 | 41 | 26 | mixed | HT | 5 |
| 8 | 300 | 51 | 20 | lytic | HT | 8 |
| 9 | 450 | 57 | 70 | mixed | both | 11 |
| 10 | 450 | 56 | 0 | mixed | HT | 14 |
| 11 | 450 | 76 | 0 | mixed | HT | 11 |
| 12 | 450 | 64 | 11 | mixed | HT | 14 |
| 13 | 600 | 54 | 15 | mixed | both | N/A |
| 14 | 600 | 48 | 21 | lytic | HT | 14 |
| 15 | 600 | 46 | 28 | mixed | HT | N/A |
| 16 | 600 | 52 | 31 | mixed | HT | 16 |

[a]HT, hormone therapy; both, cytotoxics and hormone therapy.

**Figure 1**

evidence of a dose-response effect. Since there were no significant differences in $Ca^{a+}/Cr$ ratios between the groups the data from each were combined (Figure 1b). There was a highly significant fall in ratio after one week (0.8–0.37, $P<0.01$) and a further fall in the mean value with a minimum at week 3 (week 1 versus week 3, $P<0.001$).

In a previous study (Morton *et al.*, 1988*b*) where pamidronate was given as a 2 h infusion of 30 mg each week there was a fall of the $Ca^{2+}/Cr$ ratio from 0.85 to a minimum of 0.19 units (Figure 1c). There were no significant differences in the fall of the $Ca^{2+}/Cr$ ratios of the combined 16 patients treated with oral pamidronate compared with the 16 treated with the intravenous preparation. At the end of the four week study period all patients continued to take oral pamidronate at a dose of 300 mg daily. The median subsequent follow up time was 15 weeks (range 7–21 weeks). In twelve patients the $Ca^{2+}/Cr$ ratios remained low. However, in four patients there was progression of disease in bone despite treatment and a rise of the mean ratio to pre-treatment levels (Figure 1d).

*Toxicity*

There was no gastrointestinal toxicity as doses of 150 mg and 300 mg daily. At 450 mg daily one out of four patients experienced WHO grade 1 nausea and abdominal pain for 8 days one week after starting treatment. After dose reduction to 300 mg this was abolished. At 600 mg daily one of four patients required dose reduction because of similar gastrointestinal toxicity; she tolerated 300 mg daily without further problems. Oral ulceration was not seen and there was no haematological toxicity at any dose level. Two patients on 600 mg daily, developed asymptomatic hypocalcaemia (corrected calcium 2.08 to 2.17 mmol$^{-1}$; normal range 2.2 to 2.65 mmol$^{-1}$) one week after starting treatment which persisted for one week in one patient and for two weeks in the other.

### Discussion

The bisphosphonates are absorbed poorly after oral administration. Using radiolabelled pamidronate it is estimated that, in rats, 2% of the oral dose is absorbed (Ciba-Geigy, data on file). Estimates of the absorption of another bisphosphonate (etidronate) range from 1 to 9% (Fogelman *et al.*, 1986). The effect of oral bisphosphonates on calcium release from bone may however be assayed indirectly by estimating their effect on the urinary $Ca^{2+}/Cr$ ratio. Our study indicates that oral pamidronate does significantly inhibit calcium excretion. Sufficient amounts are absorbed to give reductions in urinary calcium/creatinine ratios equivalent to given the drug at a dose of 30 mg weekly by the intravenous route.

The oral doses used were chosen to show a dose-response effect. However, the effect of 150 mg daily was not significantly different from the other three doses. It is therefore possible that much lower doses will be effective and further studies are required in order to determine the minimum effective dose. Assuming 2% oral bioavailability, 21 mg of pamidronate would be absorbed per week at a dose of 150 mg per day. This is a similar total weekly amount of pamidronate given to patients in our previous study using 30 mg per week intravenously. The reduction in calcium excretion was similar using the two routes of administration and supports the conclusions from a study on tumour-induced hypercalcaemia that the effect of pamidronate on the reduction in serum calcium levels is largely unrelated to the rate of its administration (Ralston *et al.*, 1988). In this study pamidronate was non-toxic. Only two patients developed symptoms. These were mild epigastric discomfort which subsided after the dose was lowered to 300 mg daily. Thus no patient on 300 mg per day or a lower dose developed gastrointestinal toxicity and this compares favourably with the 8% of patients who stopped using the drug because of toxicity in the series reported by van Holten-Verzantvoort *et al.* (1987).

Two patients developed mild hypocalcaemia; although asymptomatic the significance of this needs to be addressed in future studies where the drug is used for longer periods.

Pamidronate whether given orally or intravenously reduced the increased calcium excretion associated with bone metastases in all patients. However, in this study as with our previous study, using intravenously administered drug, resistance to pamidronate develops in a proportion of patients after a few weeks. Since bisphosphonates are thought to act by inhibition of tumour stimulated osteoclastic activity it is likely that they would be inactive against metastases where there is a direct bone destruction (Galasko, 1976). However, this would not explain why calcium excretion was reduced in all patients studied, albeit briefly in some.

In conclusion oral pamidronate is as effective as the intravenous preparation for reducing increased calcium excretion associated with metastatic breast cancer to bone. Future studies directed towards determining the minimum effective dose, on long-term toxicity and on the place of pamidronate combined with other therapeutic modalities in the management of patients with bone metastases are indicated.

We would like to thank Ciba-Geigy Pharmaceuticals for supplies of pamidronate and for financial support.

### References

COLEMAN, R.E., WOLL, P.J., MILES, M., SCRIVENER, W. & RUBENS, R.D. (1988). 3 Amino-1, 1-hydroxypropylidene bisphosphonate (APD) for the treatment of bone metastases from breast cancer. *Br. J. Cancer*, **58**, 621.

FLEISH, H. (1983). Bisphosphonates, mechanisms of action and clinical applications. *Bone Mineral Res. Ann.*, **1**, 319.

FOGELMAN, I., SMITH, L., MAZESS, R., WILSON, M.A., & BEVAN, J.A. (1986). Absorption of oral diphosphonate in normal subjects. *Clin. Endocrinol.*, **24**, 57.

GALASKO, C.S.B. (1976). Mechanisms of bone destruction in the development of skeletal metastases. *Nature*, **263**, 507.

MORTON, A.R., CANTRILL, J.A., CRAIG, A.E., HOWELL, A., DAVIES, M. & ANDERSON, D.C. (1988). Single dose versus daily intravenous aminohydroxypropylidene bisphosphonate (APD) for the hypercalcaemia of malignancy. *Br. Med. J.*, **296**, 811.

MORTON, A.R., CANTRILL, J.A., PILLAI, G.V., MCMAHON, A., ANDERSON, D.C. & HOWELL, A. (1988). Sclerosis of lytic bone metastases after disodium aminohydroxypropylidene bisphosphonate (APD) in patients with breast carcinoma. *Br. Med. J.*, **297**, 772.

JUNG, A., VAN OUWENALLER, C., CHANTRAINE, A. & COURVOISIER, B. (1981). Parenteral disphosphonates for treating malignant hypercalcaemia. *Cancer*, **48**, 1922.

RALSTON, S.H., ALZAID, A.A., GALLAGHER, S.J., GARDNER, M.D., COWAN, R.A. & BOYLE, I.T. (1988). Clinical experience with aminohydroxypropylidene bisphosphonate (APD) in the management of cancer-associated hypercalcaemia. *Q. J. Med.*, **258**, 825.

VAN HOLTEN-VERZANTVOORT, A., BIJVOET, O.L.M., CLETON, F.J. & 8 others (1987). Reduced morbidity from skeletal metastases in breast cancer patients during long-term bisphosphonate (APD) treatment. *Lancet*, **ii**, 983.